# *SyncMRT*: a solution to image-guided synchrotron radiotherapy for quality assurance and pre-clinical trials

**DOI:** 10.1107/S1600577522004829

**Published:** 2022-06-07

**Authors:** M. J. Barnes, J. Paino, L. R. Day, D. Butler, D. Häusermann, D. Pelliccia, J. C. Crosbie

**Affiliations:** a ANSTO Australian Synchrotron, Kulin Nation, Clayton, Victoria, Australia; b Peter MacCallum Cancer Centre, Kulin Nation, Melbourne, Victoria, Australia; c School of Science, RMIT Univeristy, Kulin Nation, Melbourne, Victoria, Australia; d Illawarra Health and Medical Research Institute, Dharawal Nation, Wollongong, New South Wales, Australia; e Centre for Medical Radiation Physics, University of Wollongong, Dharawal Nation, Wollongong, New South Wales, Australia; f Australian Radiation Protection and Nuclear Safety Agency (ARPANSA), Kulin Nation, Yallambie, Victoria, Australia; g Instruments and Data Tools, Kulin Nation, Melbourne, Victoria, Australia

**Keywords:** image guidance, radiation therapy, pre-clinical, synchrotron

## Abstract

In this work, a new image guidance system and protocols for delivering image-guided radiotherapy (IGRT) on the Imaging and Medical Beamline (IMBL) at the ANSTO Australian Synchrotron are introduced. The image guidance methods used and the resulting accuracy of tumour alignment in *in vivo* experiments are often under-reported. Image guidance tasks are often complex, time-consuming and prone to errors. If unchecked, they may result in potential mis-treatments. We introduce *SyncMRT*, a software package that provides a simple, image guidance tool-kit for aligning samples to the synchrotron beam. We have demonstrated sub-millimetre alignment using *SyncMRT* and the small-animal irradiation platform (the DynamicMRT system) on the IMBL. *SyncMRT* has become the standard for carrying out IGRT treatments on the IMBL and has been used in all pre-clinical radiotherapy experiments since 2017. Further, we introduce two quality assurance (QA) protocols to synchrotron radiotherapy on the IMBL: the Winston-Lutz test and hidden target test. It is shown that the presented QA tests are appropriate for picking up geometrical setup errors and assessing the end-to-end accuracy of the image guidance process. Together, these tools make image guidance easier and provide a mechanism for reporting the geometric accuracy of synchrotron-based IGRT treatments. Importantly, this work is scalable to other delivery systems, and is in continual development to support the upcoming veterinary radiotherapy trials on the IMBL.

## Abstract 2

A pre-clinical image guidance solution capable of achieving sub-millimetre alignment to the synchrotron beam has been implemented on the Imaging and Medical Beamline (IMBL) at the ANSTO Australian Synchrotron. Our solution, consisting of hardware, software and quality assurance protocols, has become the new standard for all image-guided radiotherapy experiments on the IMBL.

## 1. Introduction

Synchrotron microbeam radiation therapy (MRT) is a pre-clinical radiotherapy technique, first reported in the 1990s (Slatkin *et al.*, 1992 ▸). The MRT technique stemmed from the idea of exploiting the tissue sparing effects of radiation when delivered on a micrometre scale (Curtis, 1967 ▸), and expanding it to a full field of quasi-parallel micrometre-wide beamlets. Numerous MRT studies went on to demonstrate a unique tumoricidal effect, whereby a differential response was observed between normal and tumour tissue (Crosbie *et al.*, 2010 ▸; Dilmanian *et al.*, 2002 ▸; Laissue *et al.*, 1998 ▸). Since then, many studies have investigated the use of MRT to treat various tumour-bearing rodents (Trappetti *et al.*, 2021 ▸; Bouchet *et al.*, 2010 ▸; Fernandez-Palomo *et al.*, 2020*a* ▸,*b* ▸; Schültke *et al.*, 2008 ▸; Ibahim *et al.*, 2016 ▸; Yang *et al.*, 2019 ▸; Engels *et al.*, 2020 ▸; Paino *et al.*, 2021 ▸). Although these studies have reported key biological findings that solidify the clinical usefulness of MRT, some of these studies have concluded that image guidance (Engels *et al.*, 2020 ▸; Paino *et al.*, 2021 ▸) and conformal fields (Bouchet *et al.*, 2010 ▸) are necessary in order to ensure tumour coverage whilst reducing the irradiation of healthy tissues. Despite the evident need for a comprehensive image-guided radiotherapy (IGRT) solution, very few studies have detailed the image guidance protocols used and the achieved geometric treatment accuracies. Detailed reporting of image guidance protocols is required as small-animal studies become more complex and veterinary trials with larger animals approach.

In this work, we demonstrate a comprehensive IGRT solution for the small-animal irradiation platform (the DynamicMRT system) at the Imaging and Medical Beamline (IMBL) at the ANSTO Australian Synchrotron. Livingstone *et al.* previously provided a description of the DynamicMRT system and demonstrated its capabilities in a small-animal feasibility study (Livingstone *et al.*, 2017 ▸). For imaging purposes, the DynamicMRT system is equipped with an optical camera, alongside a recently installed, off-axis kilovoltage X-ray tube and flat-panel detector.

The first experiments on the DynamicMRT system utilized the optical camera to align cell flasks and rodents to the synchrotron beam via visible surface landmarks. However, in these pre-clinical studies, the information gained by simply looking at the surface was insufficient to align internal anatomy or tumour volumes to the synchrotron beam. To overcome the lack of anatomical information, image guidance with X-rays was explored. Low-dose imaging with synchrotron X-rays is complicated by the extremely high dose rates (of the order of tens to thousands of Gy s^−1^). Nevertheless, at the ID17 Biomedical beamline at the European Synchrotron Radiation Facility (ESRF), two groups were able to acquire low-dose planar X-ray images (Serduc *et al.*, 2010 ▸), and even CT images (Nemoz *et al.*, 2016 ▸), with a polychromatic synchrotron X-ray beam. A 3D alignment method was also developed for the ESRF that made use of two orthogonal 2D X-ray radiographs generated with the synchrotron beam (Donzelli *et al.*, 2016 ▸). Meanwhile, on the IMBL, another method for achieving low-dose planar X-ray images was developed using the monochromatic synchrotron beam (Pelliccia *et al.*, 2016*a* ▸), with phase-contrast capabilities (Pelliccia *et al.*, 2016*b* ▸). However, in both cases, changes were required to switch the synchrotron beam from a ‘treatment’ configuration (polychromatic) to an ‘imaging’ configuration (monochromatic). This change in beamline configuration is not only technically challenging but it is also time-consuming and has an increased risk of introducing errors into the alignment and subsequent irradiations. Additionally, the maximum synchrotron beam size at the DynamicMRT system is only 30 mm in width, meaning that larger images require the stitching of several smaller images. Thus, the kilovoltage X-ray tube is ideal for image guidance given the large beam area and low-dose imaging capabilities, and circumventing the need to make changes to the synchrotron therapy beam for imaging purposes.

The aim of this work is to extend the image guidance capabilities of the DynamicMRT system to include the X-ray tube, and provide a control platform that enables full 3D alignment. To this end, we present a software, *SyncMRT*, that solves the technical challenges faced by synchrotron IGRT and provides much needed features that streamline the image guidance process. *SyncMRT* is capable of many IGRT tasks that are commonplace in clinical workflows. *SyncMRT* can acquire radiographic images on the beamline and register them to DICOM images and radiation treatment plans. *SyncMRT* can also calculate a six degrees of freedom (6 DoF) alignment and apply it to a system with lesser degrees of freedom (for example, the DynamicMRT system). *SyncMRT* also allows for both 2D and 3D alignment based off one or two radiographs, respectively. Image orthogonality is not required where two radiographs are used for 3D alignment. For easy control of the synchrotron beamline, the hardware is grouped by function and presented in *SyncMRT* as: radiation sources, shutters, imaging detectors, positioning stages and treatment delivery apparatus. Scripting capabilities are also included to allow for non-standard functionality such as auto-breath holding during imaging and treatment.

In addition to providing accurate image guidance, we address the lack of accuracy information reported in pre-clinical IGRT studies by introducing a quality assurance (QA) programme. We demonstrate two QA protocols for assessing the accuracy of both the beamline configuration and the image guidance process using *SyncMRT*. The first protocol assesses the isocentre congruency between the treatment delivery system (the DynamicMRT system) and the synchrotron beam. The second protocol assesses the capabilities of *SyncMRT* as an image guidance system, by positioning a hidden target within the beam using anatomical information. Together, these IGRT and QA techniques serve as a unique IGRT solution for pre-clinical synchrotron MRT studies on the IMBL.

## 2. Materials and methods

### 2.1. The DynamicMRT system

**Figure 1:**
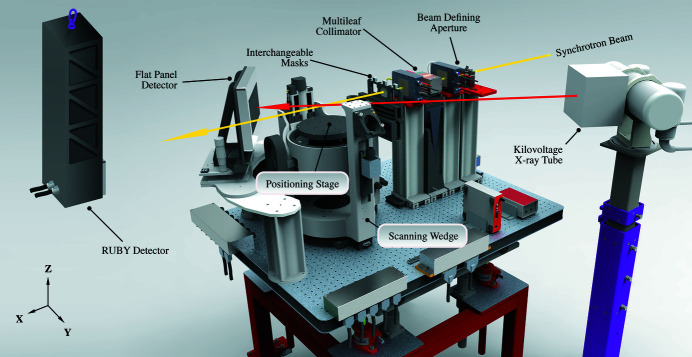
The DynamicMRT system and its main components are illustrated. The beam-shaping apparatus consists of the beam-defining aperture, the multileaf collimator and the interchangeable masks. The positioning stage is responsible for moving the sample whilst the scanning wedge (the large aluminium body) vertically moves the sample and the masks in unison. The kilovoltage X-ray tube and flat-panel detector are used for acquiring images of the sample for image guidance purposes. The IMBL coordinate system is included for reference.

Briefly, the DynamicMRT system (shown in Fig. 1 ▸) consists of a 4 DoF positioning stage, a series of beam-collimating devices, two free-air ion chambers and an off-axis kilovoltage X-ray tube and flat-panel imaging detector (Livingstone *et al.*, 2017 ▸). Additionally, the DynamicMRT system also has two visible in-room lasers (not shown) that can be set to visibly reflect the position of the synchrotron beam.

To understand the configuration process of the Dynamic­MRT system, a few key notes about the system’s capabilities are provided. Importantly, the synchrotron beam remains fixed and thus all alignment must be carried out with respect to the fixed beam position. The first component to encounter the beam is an adjustable beam-defining aperture (BDA), which limits the beam height to 0.5 mm, 1.0 mm or 2.0 mm, and the beam width to within 30 mm. The BDA is attached directly to the DynamicMRT table and cannot be moved independently. The table is only capable of moving in the vertical axis, thus, due to the fixed nature of the BDA, the beam that is delivered to the sample is set by the table height.

The second component to encounter the beam (now defined by the BDA) is the microbeam collimator. The microbeam collimator can be moved in and out of the beam vertically, and rotated about the vertical axis (*Z*). The third and final beam-shaping apparatus is the conformal mask system, which can hold up to three interchangeable masks and can be moved independently along the horizontal axis (*Y*). The positioning stage allows movement of the sample independent of the rest of the DynamicMRT system; it offers translation in (*X*, *Y*, *Z*) and vertical rotation about (*Z*). However, for irradiations, the sample and mask are designed to move together through the beam. In order to achieve this, a frame that acts as the base for both the positioning stage and the mask system is used (the ‘scanning wedge’ in Fig. 1 ▸). The scanning wedge can only move in the vertical axis (*Z*).

Finally, the off-axis imaging system is made up of two components. The first component is a 150 kV Toshiba Rotanode^TM^ cone-beam X-ray source (model E7252X), which is fixed to the ground independently of the DynamicMRT table. The second component is the Hamamatsu CsI flat-panel detector (model C9252-DK14), which is fixed to the DynamicMRT table; thus, as the table moves up and down, the geometry between the source and imaging detector is changed. This off-axis imaging system is offset from the primary synchrotron beam by 32.7°. The geometrical setup of this off-axis imaging system provides images with an effective pixel size of 160 µm × 160 µm at the isocentre and covers an area of 243.2 mm × 123.2 mm, which is sufficient for identifying bony anatomy in small animals, in a single image, for image guidance purposes.

To perform accurate IGRT with this equipment, careful configuration of each component must be performed. Additionally, correct operation of the equipment must also be considered, owing to the various coordinate systems, conventions and control mechanisms employed per-device.

### 2.2. Configuration of the IMBL and DynamicMRT system

Together, both the DynamicMRT system and the beamline can be considered an entire pre-clinical treatment system. The configuration of this system for IGRT relies on identifying the central axis of all components that define the beam shape and size as well as anything that affects positioning of the sample within the beam.

The configuration protocol for the treatment system and finding its isocentre is as follows:

(1) Define the treatment beam.

(2) Identify the isocentre of the positioning stage and align it to the treatment beam.

(3) Identify the centre of the masks and align them to the treatment beam.

(4) Configure and calibrate the off-axis imaging system.

(5) Align the in-room lasers to the isocentre.

#### 2.2.1. Defining the treatment beam

The IMBL has two types of synchrotron beams that are available for use (Stevenson *et al.*, 2017 ▸). The first available beam is a polychromatic ‘pink’ beam, with tunable energy spectra by way of filtration of the beam; this is the primary beam and it sits at a nominated height of *Z* = 0 mm on the beamline. The second beam is a monochromatic beam, generated from a double-crystal Laue monochromator (DCLM); this beam is positioned at *Z* = 20 mm (above the pink beam). Within the context of radiotherapy studies, the pink beam is typically used for the vast majority of irradiations.

A small ballbearing (0.8 mm in diameter) is placed on the positioning stage as a reference point for locating the isocentre of the treatment system. The ballbearing ultimately becomes the link between mechanical positioning and the centre of the beam. The ballbearing can be imaged using the monochromatic synchrotron beam, at any field strength, with any desired *in vacuo* filtration, providing that the beam can create good contrast for the ballbearing. The 2 mm BDA is used as this provides the largest field of view during alignment.

The rationale for using monochromatic beam for alignment is based on two assumptions that are generally regarded as acceptable. The first assumption is, given that the position of both the pink and monochromatic beams is known, we can accurately move from one beam to the other without introducing geometric errors. The second is that there is no geometrical difference between the position of beams produced at different wiggler fields and filtrations. These two assumptions infer that the geometrical stability of the beam remains fixed, whilst the dosimetric properties of the beam may be changed as desired, and have been validated experimentally (data not shown). Although it is possible to image a ballbearing with the polychromatic beam, leaving a high-intensity polychromatic beam incident on an imaging detector with only a small ballbearing in the centre, unless heavily filtered, would result in both poor image quality and damage to the imaging detector. Since the monochromatic beam has considerably less flux than the polychromatic beam and a ballbearing can be safely imaged with an imaging detector, it is favourable to configure the DynamicMRT system with the monochromatic beam. The aforementioned assumptions allow the user to configure the beam in monochromatic mode and simply switch between any desired beams for either imaging or treating after the configuration has been performed.

Typically, we choose a field strength of 3 T and 2.83 mm of copper filtration with a monochromatic energy of 45 keV since this provides both good signal in our imaging detectors and contrast in a 0.8 mm ballbearing. For imaging, the RUBY detector is used as it provides the optimal balance between a large field of view and a small pixel size (Hall *et al.*, 2013 ▸); for configuration, a pixel size of about 6 µm to 10 µm is employed.

With the imaging beam configured, the BDA (fixed to the DynamicMRT table) is then centred on the synchrotron beam. The DynamicMRT table is vertically scanned through the beam; the table has no lateral adjustment and thus any lateral offset between the synchrotron beam and the BDA cannot be accounted for. The Multileaf Collimator is then centred to the BDA. The collimator is vertically scanned and rotated (about *Z*) until each microbeam slit is fully illuminated by the synchrotron beam. It is important that the DynamicMRT table position and up-stream slit centre positions do not change, as doing so will invalidate the system’s alignment. Further, imaging with the collimator in place is impossible. Therefore, the collimator is lowered out of the beam in order to complete the configuration procedure.

#### 2.2.2. Aligning the rotation isocentre to the beam

Once the treatment beam has been defined, the mechanical rotation isocentre of the DynamicMRT system and its components must be identified. To identify the rotation isocentre of the DynamicMRT system, the ballbearing is first coarsley positioned in the crosshairs of the in-room lasers.

The RUBY detector is then used to capture a sequence of images that can be used to identify the isocentre of the DynamicMRT system. Four images are acquired of the ballbearing at −90°, 0°, 90° and 180° rotation. The difference in position of the ballbearing between axis-paired images is then calculated, and two translation motors are used to centre the ballbearing on the rotation stage. The ballbearing is then continuously imaged as it is rotated by 360° to visually observe any deviations that may present in the ballbearing position over the full range of motion. The ballbearing is now positioned at the isocentre of rotation of the DynamicMRT system.

Given that there are no tilt axes in the DynamicMRT system, the rotation axis cannot be adjusted. During acquisition of the four images, if the ballbearing rises and falls as it is rotated around then the rotation axis has become mis-aligned with the DynamicMRT system. If the rotation axis has become mis-aligned, then an obvious mechanical failure has occurred and must be fixed before proceeding.

**Figure 2:**
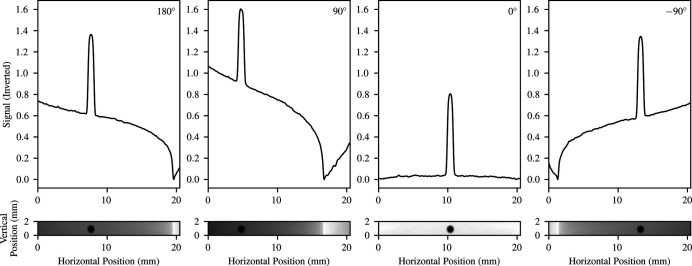
Pictured is the rotation alignment process where the ballbearing position is automatically identified using the peak attentuation in four orthogonal images over 360°. The positions of the ballbearing in each image are used to identify the centre of rotation of the DynamicMRT system.

On the IMBL, the above process is scripted so as to remove human error from this portion of the configuration process. The script captures both flood-field and dark-field images – it determines the position of the ballbearing in each view, then calculates and applies the required translations to the positioning stage. A Gaussian filter is used to remove low-level noise in the images, and horizontal line profiles are used to find the peak attenuation, representative of the ballbearing position within the beam. The script typically centres the ballbearing on the rotation isocentre to within a pixel or two over the full range of rotational motion of the stage. Thus, the accuracy of determining the rotation isocentre relies heavily on the calibrated pixel size of the detector. Typically, we achieve rotational isocentre definition to within 8 µm to 16 µm. An example image of the ballbearing aligned to the rotation isocentre of the stage is shown in Fig. 2 ▸.

#### 2.2.3. Aligning the masks

As previously mentioned, the DynamicMRT system is capable of holding three separate masks. The horizontal centre position of each mask is pre­defined, but can be adjusted as required. Each mask position is loaded on the DynamicMRT system and adjusted laterally until they are centred upon the ballbearing.

**Figure 3:**
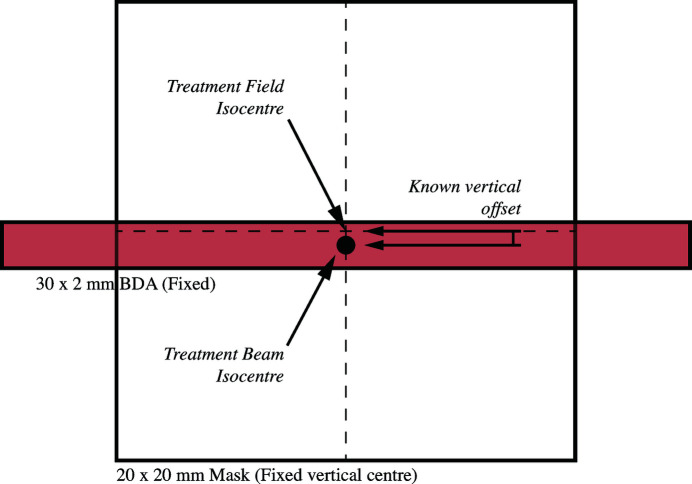
The known vertical offset between the centre of the mask and BDA is illustrated. The BDA is fixed to the DynamicMRT table and is set in height such that it is fully illuminated by the synchrotron beam. The vertical mask position is set by the scanning wedge, and, due to its small range of motion, cannot be adjusted vertically. Therefore, the ballbearing must be vertically shifted to sit within the centre of the mask, as this ultimately defines the field that is delivered to the patient.

Importantly, there exists a fixed offset between the BDA and the centre of the range of motion of the scanning frame. Thus, there is an expected vertical offset between the centre of the BDA and the centre of the masks; this offset is illustrated in Fig. 3 ▸. Recall that the DynamicMRT system has two vertical adjustment systems, one for positioning (the positioning stage) and one for treatment delivery (the scanning frame). The mechanical range of the scanning frame allows for a 2 mm beam to sit above and below a 20 mm mask by approximately 0.1 mm, therefore re-calibrating the scanning frame range to allow for the offset is not possible. Instead, using the vertical positioning capabilities of the system, the ballbearing must be adjusted to the vertical centre of the scanning frame. Consequently, if the vertical alignment is left uncorrected, it will lead to positioning offsets during irradiation, resulting in geometric mis-alignment of the tumour target.

#### 2.2.4. Calibrating the off-axis imaging system

The ballbearing is now centred on the rotation isocentre of the stage and is vertically centred about the beam-defining apparatus. The position of the ballbearing is reflective of the isocentre of the system as a whole. In order to correctly link the off-axis imaging system to the treatment beam, the beamline must be configured for pink beam again. The off-axis radiographic imaging system is now used to image the ballbearing in its final position. Note that all images that are acquired for positioning with the off-axis imaging system must include the 32.7° offset. The offset is required to rotate the object from the treatment beam position into the off-axis radiographic imaging beam position; the offset must be reversed after imaging and before treating. Two orthogonal images should be taken at 0° and 90° rotation to ensure the ballbearing position remains unchanged with rotation angle. The nominal pixel size of the off-axis X-ray system is 160 µm, whereas the alignment of the ballbearing isocentre is accurate to within 8 µm to 16 µm (about 1–2 pixels of the RUBY detector), thus no difference should be observed between the two orthogonal images acquired with the off-axis imaging system.

The centre of the ballbearing is then manually located as a pixel location in the image taken with the off-axis radiographic imaging system. This coordinate (as a pixel row, column index), in conjunction with the pixel size of the detector, can then be saved in *SyncMRT*, providing images with a spatial reference of the system’s isocentre.

#### 2.2.5. Aligning the in-room lasers

To finish the configuration, the in-room optical lasers must be aligned to the ballbearing as these will act as a coarse visual surrogate for beam position. A piece of radiochromic film can also be irradiated and used to align the lasers to.

### 2.3. Alignment with *SyncMRT*

Alignment of objects to the synchrotron beam using *SyncMRT* is performed using a point-based registration method. Registration requires information from two sources: (1) a previously acquired CT scan used to plan the treatment and (2) beamline X-ray radiographs that indicate the sample’s current position with respect to the synchrotron beam. The user is required to select a minimum of three visible landmarks in each image dataset used for registration.

To calculate the alignment, the points must first be mapped to their respective coordinate systems. The points selected in the beamline radiographs are mapped to the synchrotron coordinate system; the underlying mathematics of this process are briefly described by Jin *et al.* (2006 ▸). The points selected in the CT scan are in the DICOM coordinate system and require no mapping. These data points are then used with Horn’s method (Horn, 1987 ▸) to extract a 4 × 4 transformation matrix that would bring the patient into alignment with the synchrotron beam. Our approach is very similar to that presented by Donzelli *et al.* (2016 ▸), although since we do not have a 6 DoF alignment system (as was used in their work) we must further reduce the 6 DoF solution into a 4 DoF solution to match the capabilities of the DynamicMRT system. To achieve this, we decompose the 4 × 4 transformation matrix into components that represent each positioning motor in the DynamicMRT system; the contribution of each motor is modified until the difference between the 6 DoF and 4 DoF solutions is minimized (Barnes, 2018 ▸). This matrix decomposition technique also permits other alignment systems with different degrees of freedom (for example, 3 DoF or 7 DoF) to be used with *SyncMRT*.

### 2.4. Quality assurance programme

An image guidance quality assurance programme consisting of two QA protocols is presented. The first QA tool, a modified Winston-Lutz test (Lutz *et al.*, 1988 ▸), assesses the isocentre congruency between the DynamicMRT system and the synchrotron beam. This test should be performed after any change in beamline configuration. The second QA tool, the hidden target test, provides additional information about how accurately an object can be aligned to the synchrotron beam under image guidance.

#### 2.4.1. Modified Winston-Lutz test

Historically, a Winston-Lutz test is used to check the congruency between the imaging systems and the radiation isocentre of the primary beam on a megavoltage clinical linear accelerator (Lutz *et al.*, 1988 ▸). However, the same methodology can be used for a fixed beam with a delivery and positioning mechanism such as the DynamicMRT system on the IMBL.

To perform this test, a 0.8 mm ballbearing was placed on the DynamicMRT positioning stage and a 5 mm circular mask was placed in the mask holder. Using *SyncMRT* and the off-axis radiographic imaging system, an orthogonal pair of images of the ballbearing was acquired. These images were then used to align the ballbearing to the centre of the treatment beam. After the ballbearing was aligned, two pieces of radiochromic (EBT2) film were placed at orthogonal angles behind the ballbearing. The first film was irradiated at 0°, then the ballbearing was rotated by 90° and the second film irradiated. Irradiation times were chosen such that the film was not over-exposed and provided adequate contrast between the beam and ballbearing. The horizontal and vertical axes were marked on each piece of film enabling consistent alignment during irradiation and readout.

The film was then read out using an enclosed inverted microscope system (a Leica DMC-2900 camera and DMC-14000 positioning stage) with a pixel size of 7 µm. The images were read into the image processing software, *ImageJ* (v1.52k) (Rueden *et al.*, 2017 ▸). A Gaussian blur was applied to each image with a radius of two pixels. Both vertical and horizontal line profiles of the ballbearing position in the mask were measured. To increase the signal-to-noise ratio in the images, the average profile of 20 adjacent line profiles was used for measurement. The full width at half-maximum (FWHM) of the ballbearing and the field in the averaged line profiles were used to determine the centre of the ballbearing as a distance from the centre of the circular field. From the two orthogonal images, the 3D displacement of the ballbearing from the radiation isocentre was determined.

The readout process, including selection of the FWHM points, was repeated three times giving a standard deviation of ±0.05 mm; this is taken to be the accuracy of this readout method.

### 2.5. Hidden target test

The ‘hidden target test’ is similar to the Winston-Lutz test in Section 2.4.1, with the inclusion of an image guidance task and a more complex phantom (Lutz *et al.*, 1988 ▸). The hidden target test is an end-to-end test covering pre-treatment imaging, treatment planning, image guidance, patient positioning and treatment delivery. This test requires a phantom that has some X-ray-visible structures for alignment, and a target for treatment. A treatment plan is then generated for the phantom, and the treatment is delivered under image guidance. Film (or an imaging device) is typically used to determine the geometric accuracy of the treatment.

In this work, we used a 100 mm by 100 mm by 100 mm acrylic cube with four 3 mm ballbearings at fixed locations inside the phantom, similar to the ISO Cube (CIRS Inc, Norfolk, VA, USA) phantom. Two CT scans were acquired to investigate the reproducibility of the process and investigate any obvious effects that the CT scans had on the alignment outcomes. The CT scans were acquired on a Phillips Brilliance Big Bore CT Scanner with slice thicknesses of 1 mm and square pixel sizes of 1.17 mm. In each scan, the alignment phantom was roughly aligned to the positioning lasers. Due to the size of the phantom, the limited range of motion of the DynamicMRT system made it difficult to perform a full 3D alignment. Instead a 2D alignment was performed in the vertical and horizontal axes (*Y* and *Z*), whilst the depth (*X*) remained constant.

A CT dataset was preloaded into *SyncMRT* and one of the four ballbearings were chosen as a target location, with the remaining three ballbearings used as alignment features. Radiochromic film was then affixed to the rear of the phantom, and the phantom was coarsely positioned on the DynamicMRT system. Appropriate markings were placed on the film to ensure that alignment of the film could be determined during film readout. Two orthogonal kilovoltage X-rays were acquired with the off-axis radiographic imaging system. *SyncMRT* was then used to select the same three ‘alignment features’ (ballbearings) in the locally acquired X-ray images. Importantly, the hidden target was not used for alignment, as using it for alignment purposes would nullify the test results. The target was aligned to the synchrotron beam and a 10 mm-diameter circular field was chosen. Irradiations were then performed and the film analysed in the same manner as described in Section 2.4.1. This procedure was repeated for both CT datasets.

## 3. Results

### 3.1. SyncMRT

**Figure 4:**
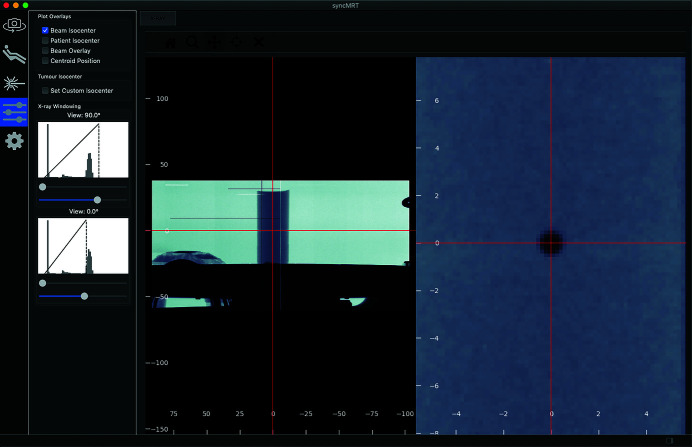
The calibration outcome for *SyncMRT* and the DynamicMRT system is shown. *SyncMRT* depicts an orthogonal pair of radiographic X-ray images containing a 0.8 mm ballbearing encased in a plastic cylinder on the DynamicMRT stage (the right-hand image has been zoomed in for illustration purposes). The ballbearing (indicative of the isocentre of the system) and the red crosshairs (representing the synchrotron beam) are accurately aligned, such that the synchrotron beam position is now known for future radiographic images. The lines and patches of dead or damaged pixels present in the images are a result of radiation damage to the imaging detector.

*SyncMRT*, pictured in Fig. 4 ▸, is a software written in Python3, and presented to the user through the Qt5 framework. The primary purpose of *SyncMRT* is to present the user with simple tools to carry out image guidance, whilst shielding the user from the complexity of the beamline. Once configured, the DynamicMRT system, the beamline and *SyncMRT* become a powerful pre-clinical IGRT system. The calibration steps outlined in Section 2.1 result in an accurate system setup, as shown by the ballbearing used for calibration in Fig. 4 ▸. When configured, all coordinate systems, control systems and apparatus are intelligently handled within *SyncMRT*. The user is presented with a set of tools that allow them to image and align a patient to the beam and deliver a prescribed treatment.

**Figure 5:**
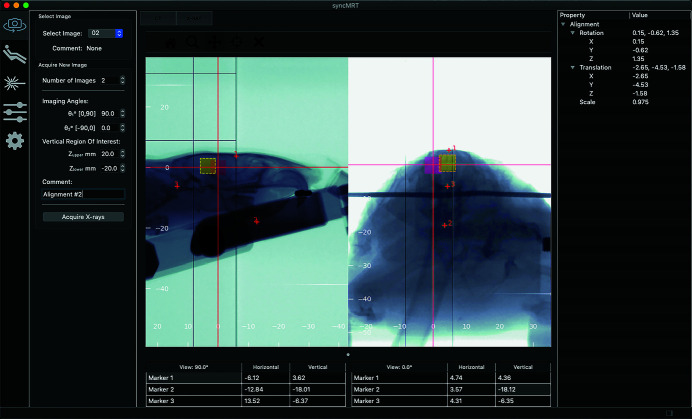
An alignment of a rodent to the synchrotron beam is shown using *SyncMRT*. The rodent, immobilized on the DynamicMRT system, is imaged using the radiographic X-ray tube at 0° and 90°. Three bony anatomical landmarks are selected in each image, allowing *SyncMRT* to calculate a movement to bring the target volume into alignment with the synchrotron beam. The red square denotes the current field position; the yellow (dotted) square denotes the desired field position (the target volume). The field overlays are a visual guide, and are only correct for the radiograph that matches the treatment angle. The lines and patches of dead or damaged pixels present in the images are a result of radiation damage to the imaging detector.

Using *SyncMRT*, users can align tumours to the synchrotron beam, even under circumstances where the tumour is not visible in the locally acquired radiographs. A clinical CT dataset can be imported into *SyncMRT* and used to co-register visible landmarks with the locally acquired radiographs. Using this point-based registration technique, the tumour position can be located and positioned in the beam. This new IGRT functionality (depicted in Fig. 5 ▸) allows complex radiotherapy treatments to be carried out on the IMBL. Additionally, *SyncMRT* and the presented configuration protocols now allow a rigorous QA programme for IGRT to be put in place.

### 3.2. QA protocol: Winston-Lutz

The Winston-Lutz test, described in Section 2.4.1, is used to determine the isocentre congruency between the synchrotron beam, the DynamicMRT system and the off-axis radiographic imaging system. The configuration of each of these components is described in Section 2.1. This configuration procedure was independently carried out by three individuals to test reproducibility. A Winston-Lutz test was performed for each configuration, resulting in three independent results; these are tabulated in Table 1 ▸.

From the results in Table 1 ▸, where the configuration procedure has been strictly followed, an isocentre congruency of sub-0.2 mm can be achieved. The large 1 mm Winston-Lutz result for ‘Beamline setup 3’ highlights that a mistake was made in the beamline configuration. Specifically, the user forgot to include the offset between the static treatment beam and the treatment field set by the mask (as illustrated in Fig. 3 ▸).

### 3.3. QA protocol: hidden target test

**Figure 6:**
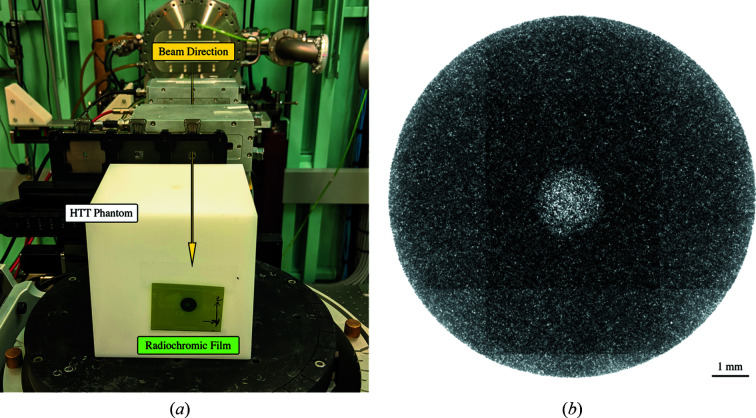
A hidden target test and its outcome are shown. (*a*) An acrylic cube phantom with four ballbearings inside; three were used for alignment and one was used as a target. (*b*) The film shows the irradiated ballbearing target within a 10 mm-diameter mask. Note that the grid-like pattern observed in the film is a result of stitching the tiles (with varying exposures) from the optical microscope images.

The hidden target test, described in Section 2.5, is used to determine how accurately an object can be aligned to the synchrotron beam under image guidance. An example of the setup for the hidden target tests is shown in Fig. 6 ▸.

After each of the two correct configurations depicted in Table 1 ▸, two hidden target tests were performed by the individual, one for each CT dataset. The results of the hidden target tests are shown in Table 2 ▸. The *Y* (horizontal) component of each hidden target test alignment was approximately 0.8 mm worse than the *Z* (vertical) component. The variation in the *Y* component was approximately 0.2 mm worse than the *Z* component. This descrepancy most likely comes down to the manual selection of points in the alignment method (described in Section 2.3).

When two radiographs are used to compute a 3D alignment, each image represents a different vertical rotational view of the subject. To combine the 2D points in each image into a 3D point requires two operations. The first operation is to combine the two vertical components, the median of these two points is used to represent the vertical (*z*) position of the 3D point. The second operation is to combine the two horizontal components, for which a mathematical routine is used to reconstruct the points in 2D to give (*x*, *y*). The 3D combined point (*x*, *y*, *z*) is then transformed into the IMBL coordinate system. Since the calculation of the vertical component is far simpler, it is more stable to varied inputs. As for the calculation of the horizontal components, these results indicate that it is sensitive to small variations in the user point selection.

Despite this, the four hidden target tests show that sub-millimetre alignment is possible, and is independent of the individual performing the tasks given the same information.

## 4. Discussion

We have demonstrated accurate image guidance on the DynamicMRT system at the IMBL with the use of a kilovoltage X-ray tube. Additionaly, we developed *SyncMRT* (the image guidance software) and devised quality assurance protocols that ensure that image guidance tasks on the IMBL meet clinical standards. This work has become the standard protocol for IGRT on the IMBL.

With careful configuration of the synchrotron beamline, Winston-Lutz tests showed <0.2 mm isocentre congruency between the synchrotron beam and positioning and imaging systems, which was of the order of our largest uncertainty (arising from the radiographic imaging detector). The Winston-Lutz test was sensitive to beamline configuration errors, as shown in ‘Beamline setup 3’ in Table 1 ▸. This sensitivity indicates that the Winston-Lutz test is an appropriate QA tool for our system. Furthermore, these results are consistent with that achieved by clinical image guidance systems on megavoltage linear accelerators. When correctly configured, the DynamicMRT system also meets the clinical standards for radiosurgery (≤1 mm) set by the AAPM TG-142 report (Klein *et al.*, 2009 ▸).

Clinically, the hidden target test is best quantified as ‘target localization error’, the measured error in target position as a result of the image guidance process. In the hidden target test, alignment and treatment of a ballbearing resulted in sub-millimetre accuracies, irrespective of beamline configuration, planning CT or operator.

Such results are made possible through *SyncMRT*, because it removes the difficulty of controlling the beamline aparatus whilst providing useful alignment tools for image guidance purposes. *SyncMRT*, the DynamicMRT system configuration and image guidance protocols presented in this paper have been used in a number of pre-clinical studies (Smyth *et al.*, 2018 ▸; Schueltke *et al.*, 2020 ▸; Trappetti *et al.*, 2021 ▸; Engels *et al.*, 2020 ▸; Paino *et al.*, 2021 ▸). In each study, the image guidance provided by *SyncMRT* allowed for the accurate alignment of tumours to the synchrotron beam. More importantly, these findings and contributions are scalable to larger delivery systems, placing synchrotron MRT one step closer towards realizing veterinary trials with large animals, such as pet dogs. Without image guidance and adequate quality assurance protocols, errors in alignment and treatment delivery cannot be eliminated. To our knowledge, this study is the first comprehensive image guidance solution for synchrotron radiotherapy trials.

Previously, image guidance on the IMBL was limited to 2D ‘click and move’ capabilities; one such example was presented by Pelliccia *et al.* (2016*a* ▸). A single image was presented to the user and the target position was identified via a mouse click; the chosen target location was then moved into the beam. Whilst suitable for simplistic treatments (*i.e.* a single field with a visible tumour location), their protocol did not allow for treatment plans or non-visible tumours. Meanwhile, on ID17 at the ESRF, Donzelli *et al.* (2016 ▸) presented a 3D alignment protocol that required two X-ray images. The alignment protocol utilized externally placed, X-ray visible fiducial markers for alignment registration; the position of the target relative to the fiducial markers was known from CT imaging and basic treatment planning. In both IGRT solutions (at the IMBL and ID17), image guidance was limited to use of the synchrotron beam for X-ray imaging. Although novel imaging techniques were developed at each facility (Serduc *et al.*, 2010 ▸; Pelliccia *et al.*, 2016*b* ▸), switching between synchrotron beam configurations for imaging and treatment is both time-consuming and prone to introducing errors. Through the use of the kilovoltage X-ray tube, we have eliminated the need to change beamline configurations whilst being able to acquire high-quality images for alignment within a matter of seconds.

To our knowledge, no previous work has included routine quality assurance protocols aimed at assessing the accuracy of, and picking up errors in, the treatment delivery system, despite several well designed phantom tests being carried out (Donzelli *et al.*, 2016 ▸; Paino *et al.*, 2021 ▸). In synchrotron radiotherapy, the beamline is configured from the ground-up at the beginning of each experiment; as such, all the components in the beamline must be re-configured for radiotherapy purposes. In a clinical environment, QA tests are performed on daily, monthly or yearly schedules depending on the type of test (Bissonnette *et al.*, 2012 ▸; Klein *et al.*, 2009 ▸); additionally, when a critical component is modified or replaced, QA tests are performed immediately to ensure the correct operation of the machine before its return to clinical service. Thus, for synchrotron radiotherapy, rigorous QA is required at the beginning of each experiment, and appropriate QA should be carried out throughout the experiment to ensure correct operation of the beamline.

We propose that the Winston-Lutz test (presented in Section 2.4.1) be carried out after configuration of the beamline for a radiotherapy experiment. The hidden target test (presented in Section 2.5) acts as a treatment simulation and should also be carried out at least once prior to each group of irradiations, ensuring consistency in the experimental setup and that all control systems are operating as expected. These tests take a matter of minutes and are capable of highlighting unexpected errors that may lead to mis-treatment.

Although *SyncMRT* has been used in several experiments over the past four years, only one study (Paino *et al.*, 2021 ▸) has presented and collected results for a QA protocol similar to those described in this paper. As such, there are no existing studies, published or unpublished, that we can draw data from. This further highlights the need to collect such data in all future studies on the IMBL.

Additionally, the presented QA protocols require further refinement for continued use. For example, the hidden target test should incorporate at least one treatment field that requires a large rotation component (≥15°). A repeated study with larger sample numbers would also provide insights into the reproducibility of the DynamicMRT and *SyncMRT* IGRT systems. A database of QA outcomes should also be kept to assess the efficacy of this work over a long-term period. With a database of QA outcomes and phantom studies, the geometrical uncertainty contribution to treatment planning margins for synchrotron MRT could, over time, be obtained.

Whilst *SyncMRT* is under on-going development, there are some obvious limitations of the software that will become the focus of future work. Firstly, the treatment field overlays, as depicted in Fig. 5 ▸, are simply projected onto the field isocentre coordinates. These overlays are correctly displayed on an image with the same angular projection as the treatment field. However, if the angular projections are mis-matched, the field overlays will not be true overlays, although they are still useful indications of where the field is located.

Furthermore, our image registration algorithm, like the algorithm presented by Donzelli *et al.* (2016 ▸), relies on a user manually selecting landmarks in each image, the combination of which ultimately defines the position of the patient. This method is subject to variation, and thus alignment results will vary from experiment to experiment. A study investigating the effects of marker placement (more commonly known as ‘fiducial localization error’) and its effect on correctly localizing the target (‘target registration error’) should be performed. Ideally, a method in which landmarks are automatically chosen could result in more accurate and more consistent alignment outcomes.

The work presented in this study provides a foundation for high-quality image guidance in pre-clinical studies. Recently, Paino *et al.* used *SyncMRT* and the quality assurance protocols described in this work to develop a comprehensive image guidance and treatment delivery protocol for the irradiation of tumour-bearing rats receiving MRT on the IMBL (Paino *et al.*, 2021 ▸; Engels *et al.*, 2020 ▸). In their work, they explored the effects of CT reconstruction algorithms, X-ray imaging doses (from both CT and locally acquired images with the kilovoltage X-ray tube) and image guidance and treatment delivery accuracies. For quality assurance, they 3D-printed a rat-skull with a ballbearing target and, using this phantom, performed a hidden target test prior to each batch of irradiations. Histology of the treated rats demonstrated that the tumours were indeed centred within the radiation field. These works by Paino *et al.* and Engels *et al.* highlight the important role of *SyncMRT* and the devised QA protocols in achieving accurate image-guided treatments in synchrotron radiotherapy, especially given the recent increase of *in vivo* experiments on the IMBL. They also show the usefulness of the hidden target test when a more appropriate alignment phantom is used.

## 5. Conclusion

An image guidance solution for small-animal irradiations has been successfully implemented on the IMBL at the ANSTO Australian Synchrotron using the *SyncMRT* IGRT software and the DynamicMRT system. Since its first successful use in December 2017, *SyncMRT* has become the standard for carrying out image guidance on the beamline and has been used in every live-animal radiotherapy experiment since. The modified Winston-Lutz test has been shown to be an invaluable tool for assessing the configuration accuracy of the system as a whole and should be used at the beginning of every IGRT experiment on the IMBL. The hidden target test also serves as a useful end-to-end QA test of the entire IGRT workflow. Finally, *SyncMRT* provides many useful capabilities, and. when paired with the DynamicMRT system, can be configured to achieve sub-millimetre positioning accuracies. The software and protocols presented are in continual development. We anticipate that greater clinical functionality will be required by users in future experiments on the IMBL.

## Figures and Tables

**Table 1:** Winston-Lutz QA outcomes for three beamline setups The distance of the ballbearing to the beam isocentre was captured via radiochromic film in the *X*, *Y* and *Z* axes. Measurement uncertainties (as described in Section 2.4.1) are shown for each axis; the three errors are combined in quadrature to give the total uncertainty in the 3D measurement.

	Distance to isocentre (mm)			
	*X* (±0.050)	*Y* (±0.050)	*Z* (±0.050)	3D (±0.087)
Beamline setup 1	0.013	0.037	0.167	0.171
Beamline setup 2	0.041	0.032	0.049	0.074
Beamline setup 3†	0.331	0.543	0.780	1.007

† This result highlights an error in the beamline setup.

**Table 2:** The alignment accuracy of four hidden target tests are presented for the first two beamline setups and two different CT datasets The distance of the ballbearing to the beam isocentre was captured via radiochromic film in the *Y* and *Z* axes. Measurement uncertainties (as described in Section 2.4.1) are shown for each axis; the two errors are combined in quadrature to give the total uncertainty in the 2D measurement.

	Distance to isocentre (mm)		
Configuration	*Y* (±0.050)	*Z* (±0.050)	2D (±0.071)
Beamline setup 1, CT dataset 1	0.820	0.017	0.820
Beamline setup 1, CT dataset 2	0.393	0.008	0.394
Beamline setup 2, CT dataset 1	0.773	0.145	0.825
Beamline setup 2, CT dataset 2	1.022	0.161	1.071
